# Supplementary material for: Who feels like they belong? Personality and belonging in college
Source: PLoS One. 2024 Jan 17;19(1):e0295436. doi: 10.1371/journal.pone.0295436 (PMC10793887; doi:10.1371/journal.pone.0295436)
Supplement: S1 File — (DOCX) [file pone.0295436.s001.docx]

**Supplemental Material for**

**“Who feels like they belong? Personality and belonging in college”**

[Author’s Names - Blinded for Peer Review]

*This supplement provides supplemental analyses beyond those included in the main manuscript.*

Table of Contents:

[**Additional Analyses 2**](#_i06h8q2ite0a)

[**Preregistered analyses regarding “personality change” 2**](#_t2xme5j6oaje)

[Mean-level personality differences between T1 and T2 2](#_l9c1u1h6ko68)

[Table S1: Mean-level personality change over the first year of college. 3](#_tin7l6i7gjd7)

[Personality difference and T2 belonging 4](#_b8dpnyaj6mut)

[Table S2: Personality change predicting T2 belonging. 4](#_wvqpueh40d5n)

[**Preregistered exploratory analyses 6**](#_9lwtc2bcace)

[Personality and belonging uncertainty 6](#_63jmbzshrk3f)

[T1 personality and T2 belonging uncertainty 6](#_cuhg5j6xyayz)

[Table S3: T1 personality predicting T2 belonging uncertainty. 7](#_uuvdjj9al05d)

[T2 personality and T2 belonging uncertainty 8](#_j0hstkihirwu)

[Table S4: T2 personality predicting T2 belonging uncertainty. 9](#_e53cbca6hhd4)

[T2 belonging uncertainty compared to T2 belonging 10](#_1rgep1m7kpo1)

[Similarities 10](#_pwialxlpzvza)

[Differences 11](#_n4drbmo4rhhz)

[Moderation by intervention condition 12](#_axvhnyexn2yo)

[Table S5: Personality predicting T2 belonging by intervention condition. 12](#_71ufal5bp70m)

[Table S6: Moderations by intervention condition. 14](#_ihlf59ycfr7t)

[**Robustness analyses using restricted inclusion criteria 15**](#_9z39npee642t)

[T1 personality and T2 belonging in restricted sample 16](#_14thmcb711w)

[Table S7: T1 personality predicting T2 belonging in restricted sample. 16](#_vhsen3vyk9wi)

[T2 personality and T2 belonging in restricted sample 17](#_v6ofa5g0xsx6)

[Table S8: T2 personality predicting T2 belonging in restricted sample. 18](#_4zt5rns768m7)

[Restricted sample compared to manuscript sample 19](#_f6zmpu9unt2r)

[Similarities 19](#_bx5fqc2mdyxs)

[Differences 20](#_w79f0zm537ep)

[**References 21**](#_swij82lppj8f)

# **Additional Analyses**

Here we present additional analyses beyond those included in the main manuscript. The analyses fall into three categories: (1) preregistered analyses that reviewers suggested be removed from the main text, (2) preregistered exploratory analyses, and (3) robustness checks for our primary analyses.

# **Preregistered analyses regarding “personality change”**

Originally, we were interested in students’ personality change in the transition to college and how personality change may be related to belonging. However, during the peer review process, a reviewer recommended that these analyses be removed from the main mainscript and reported here in the Supplemental Material instead. They raised reasonable concerns about whether differences in personality might be due to changes in assessment context rather than “true” personality change and whether brief measures of personality are appropriate for examining personality change.

We include the relevant analyses here for transparency and to encourage research on this topic. We have adjusted our language when reporting the results of analyses to talk about personality *differences* rather than personality change.

## **Mean-level personality differences between T_1_ and T_2_**

We preregistered a research question regarding students’ mean-level personality change from the beginning of their first year of college (Time 1; T_1_) to the end of their first year of college (Time 2; T_2_).

Table S1 reports the mean and standard deviation, as well as the standardized mean change, for each personality dimension at T_1_ and T_2_. It also reports the percentage of the sample who, at the individual level, (a) decreased, (b) increased, and (c) stayed the same on each dimension between these two time points. Only students who reported personality at both T_1_ and T_2_ were included in these analyses.

A first observation is simply that, across each personality dimension, only about a third of students are reporting stability over time. Second, of students who report differences over time within a dimension, some are rating their personalities as higher over time while others are rating their personalities as lower over time. We emphasize this to suggest that the mean differences, discussed below, likely mask considerable heterogeneity.

That said, on average, students reported less extraversion, agreeableness, and conscientiousness over the course of the first year of college. These changes were small in magnitude with Cohen’s *d*s ranging from -0.11 to -0.19. Alternatively, neuroticism slightly increased from T_1_ to T_2_ by 0.1 standard score units. All of the personality dimensions, with the exception of openness, showed significant change in means, based on paired samples t-test [1]. These analyses were performed in R [2].

### **Table S1: Mean-level personality change over the first year of college.**

| **Dimension** | **T1** | | **T2** | | **Mean Change**  **Score** | **Mean Change**  **(Cohen's d)** | **Individual-level Change** | | |
| --- | --- | --- | --- | --- | --- | --- | --- | --- | --- |
|  | **Mean** | **Standard Deviation** | **Mean** | **Standard Deviation** |  |  | **Decreased** | **No Change** | **Increased** |
| **Extroversion** | 3.25 | 1.05 | 3.14 | 1.05 | -0.11*** | -0.11 | 40% | 29% | 30% |
| **Neuroticism** | 3.05 | 1.00 | 3.15 | 1.02 | 0.10*** | 0.10 | 33% | 27% | 41% |
| **Agreeableness** | 3.85 | 0.81 | 3.70 | 0.88 | -0.15*** | -0.17 | 41% | 29% | 29% |
| **Conscientiousness** | 3.82 | 0.79 | 3.66 | 0.83 | -0.15*** | -0.19 | 42% | 32% | 26% |
| **Openness** | 3.61 | 0.92 | 3.62 | 0.92 | 0.01 | 0.01 | 34% | 30% | 36% |

*N* = 1,647 for extraversion, agreeableness, and openness. *N* = 1,646 for neuroticism and conscientiousness.

*p < .05. **p < .01. ***p < .001.

## **Personality difference and T_2_ belonging**

We had also preregistered a research question regarding the relationship between personality change and belonging. However, as we explained above, reviewer feedback led us to remove the discussion of personality change (or difference) from the main manuscript and, thus, we also removed the discussion of the relationship between personality difference and T_2_ belonging. We include the relevant analyses here for transparency and to encourage research on how personality change over time relates to students’ feelings of belonging. This is of particular relevance given a growing interest in personality change interventions [3-5].

In the personality-only model, only students’ difference in extraversion was associated with their belonging at the end of their first year (Table S2). Specifically, greater reported extraversion at T_1_ relative to T_2_ was positively associated with T_2_ belonging; the greater a student’s increase in reported extraversion over their first year of college, the greater their sense of belonging at the end of that year. This model explained 1.6% of the variance in T_2_ belonging (R^2^ = .016, *F*(5, 1640) = 6.492, *p* < .001).

### **Table S2: Personality change predicting T_2_ belonging.**

|  | **Personality-only** | | **Preferred** | | **Covariate-only** | |
| --- | --- | --- | --- | --- | --- | --- |
|  | ***B***  ***(SE)*** | ***95% CI*** | ***B***  ***(SE)*** | ***95% CI*** | ***B***  ***(SE)*** | ***95% CI*** |
| **Extraversion** | .114***  (.030) | [.056, .172] | .125***  (.029) | [.068, .181] | - | - |
| **Neuroticism** | -.064*  (.028) | [-.119, -.009] | -.053  (.028) | [-.107, .001] | - | - |
| **Agreeableness** | .068*  (.029) | [.012, .125] | .048  (.029) | [-.008, .104] | - | - |
| **Conscientiousness** | .009  (.033) | [-.056, .073] | .012  (.032) | [-.051, .075] | - | - |
| **Openness** | .002  (.027) | [-.050, .054] | -.009  (.026) | [-.060, .042] | - | - |
| **Black** | - | - | -.537***  (.088) | [-.709, -.365] | -.556***  (.088) | [-.729, -.383] |
| **Asian** | - | - | -.344***  (.091) | [-.524, -.165] | -.334***  (.092) | [-.515, -.154] |
| **Hispanic** | - | - | -.139  (.102) | [-.338, .061] | -.153  (.103) | [-.354, .048] |
| **Native** | - | - | -.365  (.284) | [-.923, .193] | -.415  (.286) | [-.976, .146] |
| **Multiracial** | - | - | -.740  (.936) | [-2.576, 1.097] | -.714  (.944) | [-2.566, 1.137] |
| **Other Race** | - | - | -.328***  (.099) | [-.522, -.134] | -.313**  (.099) | [-.508, -.117] |
| **Unspecified Race** | - | - | .103  (.664) | [-1.200, 1.406] | -.035  (.669) | [-1.347, 1.277] |
| **Man** | - | - | .018  (.050) | [-.079, .116] | .005  (.050) | [-.093, .103] |
| **Other Gender** | - | - | -.256  (.190) | [-.629, .116] | -.293  (.191) | [-.667, .082] |
| **First-Generation Student** | - | - | -.045  (.056) | [-.154, .065] | -.029  (.056) | [-.139, .082] |
| **Unknown Generation Status** | - | - | -.190  (.274) | [-.728, .348] | -.220  (.276) | [-.761, .321] |
| **Adjusted R²** | 0.016 | | 0.068 | | 0.053 | |
| ***N* Observations** | 1,646 | | 1,646 | | 1,646 | |

The reference category is White, continuing-generation women in the control condition. To economize on space, we do not report the dummy variables for each school and condition (available from authors upon request).

*p < .05. **p < .01. ***p < .001.

In our preferred model which controlled for school and personal characteristics, the positive relationship between the difference in extraversion and T_2_ belonging held (Table S2). This model explained 6.8% of the variance in T_2_ belonging (R^2^= .068, *F*(24, 1621) = 6.016, *p* < .001). In addition, students who identified racial-ethnically as Black, Asian, or “Other” also reported lower belonging compared to White students.

Our preferred model that included the covariates in addition to personality had greater predictive validity than the covariate-only model (*F*(5, 1626) = 6.235, *p* < .001). Including personality in the model explained an additional 1.5% of the variance in students’ T_2_ belonging over and above the covariates.

# **Preregistered exploratory analyses**

## **Personality and belonging uncertainty**

In addition to our primary hypotheses with belonging as the outcome, we preregistered parallel analyses with belonging uncertainty--defined by Walton et al. [6] as a “a broad-based hypothesis that ‘people like me do not belong here.’”--as the outcome. Although belonging and belonging uncertainty are modestly to highly correlated [7] and researchers have often collapsed them [6, 8], they are conceptually distinct. Therefore, we present the analyses for belonging uncertainty here. Belonging uncertainty was evaluated using a single item (“When you think about [school name], how often, if ever, do you wonder: ‘Maybe I don’t belonging here’?”) on a 5-point scale ranging from 1 (*always*) to 5 (*never*). We reverse-scored the item so that higher values represent greater uncertainty about belonging.

### **T_1_ personality and T_2_ belonging uncertainty**

In our preferred model which controlled for school and personal characteristics, neuroticism and agreeableness at the beginning of students’ first year were associated with their belonging uncertainty at the end of their first year (Table S3). Specifically, T_1_ neuroticism was positively associated with T_2_ belonging uncertainty; the higher a student’ neuroticism during the summer before college, the higher their sense of belonging uncertainty at the end of their first year of college. In contrast, T_1_ agreeableness was negatively associated with T_2_ belonging uncertainty; the lower a student’ agreeableness during the summer before college, the higher their sense of belonging uncertainty at the end of their first year of college. This model explained 5.4% of the variance in T_2_ belonging uncertainty (R^2^= .054, *F*(26, 2108) = 5.652, *p* < .001). In addition, students who identified racial-ethnically as Black reported higher T_2_ belonging uncertainty compared to White students.

#### **Table S3: T_1_ personality predicting T_2_ belonging uncertainty.**

|  | **Personality-only** | | **Preferred** | | **Covariate-only** | |
| --- | --- | --- | --- | --- | --- | --- |
|  | ***B***  ***(SE)*** | ***95% CI*** | ***B***  ***(SE)*** | ***95% CI*** | ***B***  ***(SE)*** | ***95% CI*** |
| **Extraversion** | -.038  (.021) | [-.079, .002] | -.024  (.021) | [-.065, .017] | - | - |
| **Neuroticism** | .080***  (.021) | [.039, .122] | .096***  (.022) | [.052, .139] | - | - |
| **Agreeableness** | -.107***  (.026) | [-.157, -.056] | -.120***  (.026) | [-.171, -.069] | - | - |
| **Conscientiousness** | -.043  (.027) | [-.095, .009] | -.019  (.027) | [-.072, .034] | - | - |
| **Openness** | -.003  (.022) | [-.047, .041] | -.020  (.023) | [-.065, .024] | - | - |
| **Black** | - | - | .432***  (.082) | [.271, .593] | .366***  (.082) | [.204, .527] |
| **Asian** | - | - | .112  (.064) | [-.013, .237] | .135*  (.063) | [.010, .259] |
| **Hispanic** | - | - | .093  (.097) | [-.097, .284] | .073  (.098) | [-.119. .264] |
| **Native** | - | - | .395  (.280) | [-.154, .943] | .358  (.283) | [-.198, .913] |
| **Multiracial** | - | - | .890  (.926) | [-.927, 2.706] | .826  (.936) | [-1.010, 2.662] |
| **Other Race** | - | - | .125  (.090) | [-.050, .301] | .133  (.091) | [-.045, .311] |
| **Unspecified Race** | - | - | -.079  (.415) | [-.893, .734] | -.046  (.420) | [-.870, .778] |
| **Man** | - | - | -.024  (.046) | [-.115, .067] | -.061  (.045) | [-.149, .028] |
| **Other Gender** | - | - | -.157  (.156) | [-.463, .149] | -.107  (.157) | [-.415, .200] |
| **First-Generation Student** | - | - | -.023  (.049) | [-.118, .072] | -.020  (.049) | [-.117, .076] |
| **Unknown Generation Status** | - | - | .070  (.242) | [-.405, .544] | .061  (.244) | [-.418, .540] |
| **Adjusted R²** | 0.025 | | 0.054 | | 0.028 | |
| ***N* Observations** | 2,135 | | 2,135 | | 2,135 | |

The reference category is White, continuing-generation women in the control condition. To economize on space, we do not report the dummy variables for each school and condition (available from authors upon request).

*p < .05. **p < .01. ***p < .001.

Our preferred model that included the covariates in addition to personality had greater predictive validity than the covariate-only model (*F*(5, 2113) = 12.392, *p* < .001). Including personality in the model explained an additional 2.6% of the variance in students’ T_2_ belonging uncertainty over and above the covariates.

### **T_2_ personality and T_2_ belonging uncertainty**

In our preferred model which controlled for school and personal characteristics, neuroticism, extraversion, agreeableness, and conscientiousness at the end of students’ first year were associated with their belonging uncertainty at the end of their first year (Table S4). Specifically, T_2_ neuroticism was positively associated with T_2_ belonging uncertainty; the higher a students’ neuroticism at the end of their first year of college, the higher their sense of belonging uncertainty at that same time. In contrast, T_2_ extraversion, T_2_ agreeableness, and T_2_ conscientiousness were each negatively associated with T_2_ belonging uncertainty; the lower a student’s extraversion, agreeableness, or conscientiousness at the end of their first year of college, the higher their sense of belonging uncertainty at that same time. This model explained 9.6% of the variance in T_2_ belonging uncertainty (R^2^= .096, *F*(26, 4234) = 18.450, *p* < .001). In addition, Black, Asian, Hispanic, Native, and Multiracial students reported higher T_2_ belonging uncertainty compared to their White peers.

#### **Table S4: T_2_ personality predicting T_2_ belonging uncertainty.**

|  | **Personality-only** | | **Preferred** | | **Covariate-only** | |
| --- | --- | --- | --- | --- | --- | --- |
|  | ***B***  ***(SE)*** | ***95% CI*** | ***B***  ***(SE)*** | ***95% CI*** | ***B***  ***(SE)*** | ***95% CI*** |
| **Extraversion** | -.080***  (.014) | [-.107, -.052] | -.074***  (.014) | [-.102, -.047] | - | - |
| **Neuroticism** | .105***  (.015) | [.076, .134] | .122***  (.015) | [.092, .152] | - | - |
| **Agreeableness** | -.155***  (.017) | [-.189, -.122] | -.147***  (.017) | [-.181, -.114] | - | - |
| **Conscientiousness** | -.097***  (.018) | [-.132, -.062] | -.070***  (.018) | [-.105, -.035] | - | - |
| **Openness** | -.008  (.016) | [-.039, .022] | -.016  (.015) | [-.047, .014] | - | - |
| **Black** | - | - | .322***  (.053) | [.218, .426] | .293***  (.054) | [.187, .400] |
| **Asian** | - | - | .127*  (.057) | [.015, .239] | .156**  (.059) | [.041, .271] |
| **Hispanic** | - | - | .201**  (.063) | [.076, .325] | .202**  (.065) | [.074, .331] |
| **Native** | - | - | .357*  (.173) | [.018, .695] | .440*  (.178) | [.091, .789] |
| **Multiracial** | - | - | .251**  (.083) | [.088, .414] | .264**  (.086) | [.096, .432] |
| **Other Race** | - | - | .026  (.069) | [-.109, .161] | .003  (.071) | [-.136, .142] |
| **Unspecified Race** | - | - | -0.244  (.294) | [-.819, .332] | -.070  (.303) | [-.663, .524] |
| **Man** | - | - | .036  (.034) | [-.030, .102] | .003  (.033) | [-.062, .068] |
| **Other Gender** | - | - | .057  (.107) | [-.154, .267] | .180  (.111) | [-.037, .396] |
| **First-Generation Student** | - | - | .041  (.036) | [-.029, .112] | .045  (.037) | [-.027, .118] |
| **Unknown Generation Status** | - | - | 0.108  (.064) | [-.018, .234] | .123  (.066) | [-.007, .253] |
| **Adjusted R²** | 0.066 | | 0.096 | | 0.035 | |
| ***N* Observations** | 4,261 | | 4,261 | | 4,261 | |

The reference category is White, continuing-generation women in the control condition. To economize on space, we do not report the dummy variables for each school and condition (available from authors upon request).

*p < .05. **p < .01. ***p < .001.

Our preferred model that included the covariates in addition to personality had greater predictive validity than the covariate-only model (*F*(5, 4239) =58.192, *p* < .001). Including personality in the model explained an additional 6.1% of the variance in students’ T_2_ belonging uncertainty over and above the covariates.

### **T_2_ belonging uncertainty compared to T_2_ belonging**

These exploratory analyses examining the relationship between personality and T_2_ belonging uncertainty are qualitatively similar to the results reported in the main manuscript that examine personality and T_2_ belonging, with a few minor differences. We summarize the similarities and differences below. .

#### **Similarities**

In both the analyses focused on T_1_ personality and T_2_ belonging and the analyses concerned with T_1_ personality and T_2_ belonging uncertainty we see similar results regarding T_1_ agreeableness and T_1_ neuroticism. First, we found that T_1_ agreeableness was positively associated with T_2_ belonging, while T_1_ neuroticism was negatively associated with T_2_ belonging. We also found that T_1_ agreeableness was negatively associated with T_2_ belonging uncertainty, while T_1_ neuroticism was positively associated with T_2_ belonging uncertainty. These results effectively mirror each other; the higher a students’ agreeableness or the lower their neuroticism during the summer before college, the higher their sense of belonging and the lower their sense of belonging uncertainty at the end of their first year of college. In addition, T_1_ openness was found to be nonsignificant in relation to both T_2_ belonging and T_2_ belonging uncertainty.

The results of the analyses on the association between T_2_ personality and T_2_ belonging and the analyses on the association between T_2_ personality and T_2_ belonging uncertainty show almost identical results. The results in the manuscript report that T_2_ extraversion, T_2_ agreeableness, and T_2_ conscientiousness were each positively associated with T_2_ belonging, while T_2_ neuroticism was negatively associated with T_2_ belonging. The results shown in the supplement report that T_2_ extraversion, T_2_ agreeableness, and T_2_ conscientiousness were each negatively associated with T_2_ belonging uncertainty, while T_2_ neuroticism was positively associated with T_2_ belonging uncertainty. These results describe the same patterns; the higher a students’ extraversion, agreeableness, or conscientiousness or the lower their neuroticism, the higher their sense of belonging and the lower their sense of belonging uncertainty at the end of their first year of college.

#### **Differences**

The results regarding T_1_ personality reported in the manuscript and those reported here are different in two ways. First, in the T_2_ belonging analyses, T_1_ extraversion was positively associated with T_2_ belonging (*b* = .104, *se* = .021, *t* = 5.025, *p* < .001). However, in the T_2_ belonging uncertainty analyses, we found that the association between T_1_ extraversion and T_2_ belonging uncertainty was nonsignificant (*b* = -.024, *se* = .021, *t* = -1.149, *p* = .251). Second, we found a marginal effect such that T_1_ conscientiousness was positively associated with T_2_ belonging (*b* = .045, *se* = .027, *t* = 1.671, *p* = .095). In contrast, T_1_ conscientiousness was found to be nonsignificant in association with T_2_ belonging uncertainty (*b* = -.019, *se* = .027, *t* = -.712, *p* = .477). Thus, T_1_ extraversion and T_1_ conscientiousness matter less in association with T_2_ belonging uncertainty than they do in association with T_2_ belonging.

Another important difference between the manuscript results and those reported here is that personality explains less variance in the T_2_ belonging uncertainty analyses than in the T_2_ belonging analyses. Including T_1_ personality in the model explained an additional 4.7% of the variance in students’ T_2_ belonging, while including T_1_ personality in the model explained an additional 2.6% of the variance in T_2_ belonging uncertainty. Also, including T_2_ personality in the model explained an additional 8.6% of the variance in students’ T_2_ belonging, while including T_2_ personality in the model explained an additional 6.1% of the variance in T_2_ belonging uncertainty

## **Moderation by intervention condition**

As mentioned in the main manuscript, participants of the original, overarching study were assigned to two different versions of a social belonging intervention (referred to as “Standard” or “Customized”) or a control condition on a randomized basis [9]. Students in all conditions saw overview information, read stories about the transition to college, wrote an essay, and then completed survey and demographic questions. The content of the stories and the essay prompt varied by condition. Please refer to Walton et al. [6] for more details.

To ease interpretation and ensure robustness, we created indicator variables (1/0) to refer to the various conditions–(1) control condition, (2) standard condition only, (3) customized condition only, and (4) standard or customized condition. We then replicate our main regression models examining T_1_ and T_2_ personality dimensions predicting T_2_ belonging for each of these conditions separately (Table S5).

### **Table S5: Personality predicting T_2_ belonging by intervention condition.**

|  | **T2 Belonging** | | | |
| --- | --- | --- | --- | --- |
|  | **Control** | **Standard** | **Customized** | **Standard or Customized** |
| **T1 Extraversion** | .061  (.035) | .090*  (.036) | .165***  (.038) | .129***  (.026) |
| **T1 Neuroticism** | -.035  (.038) | -.031  (.038) | -.127**  (.040) | -.078**  (.027) |
| **T1 Agreeableness** | .110*  (.045) | .187***  (.044) | .168***  (.047) | .175***  (.032) |
| **T1 Conscientiousness** | .071  (.046) | .004  (.047) | .036  (.049) | .028  (.034) |
| **T1 Openness** | -.026  (.039) | -.011  (.039) | .039  (.041) | .022  (.028) |
| **T2 Extraversion** | .131***  (.024) | .178***  (.025) | .187***  (.025) | .183***  (.017) |
| **T2 Neuroticism** | -.079**  (.027) | -.073**  (.026) | -.097***  (.027) | -.084***  (.019) |
| **T2 Agreeableness** | .182***  (.030) | .168***  (.030) | .144***  (.029) | .153***  (.021) |
| **T2 Conscientiousness** | .047  (.031) | .069*  (.031) | .034  (.031) | .051*  (.022) |
| **T2 Openness** | .010  (.027) | .032  (.027) | .023  (.027) | .029  (.019) |
| ***N* Observations (T1)** | 805 | 652 | 680 | 1,332 |
| ***N* Observations (T2)** | 1,418 | 1,409 | 1,435 | 2,844 |

Standard errors in parentheses. Each column reports the coefficient of the personality dimension shown in row A predicting T2 belonging by condition from separate, covariate-adjusted, regressions. The reference category is White, continuing-generation women in the control condition. To economize on space, we do not report the dummy variables on college.

*p < .05. **p < .01. p*** < .001.

First, we find that T_2_ extraversion, T_1_ agreeableness and T_2_ agreeableness are each positively associated with T_2_ belonging across each intervention condition. Additionally, T_2_ neuroticism was found to be negatively associated with T_2_ belonging across each intervention condition. The results for the other personality dimensions were less consistent and precise. Specifically, T_1_ extraversion was positively associated with T_2_ belonging across all intervention conditions except the control condition, T_1_ conscientiousness was positively associated with T_2_ belonging only in the standard and standard or customized conditions, and T_2_ neuroticism was negatively associated with T_2_ belonging only in the customized and standard or customized conditions. Lastly, associations between (a) T_1_ conscientiousness, (b) T_1_ openness, (c) T_2_ openness and T_2_ belonging are nonsignificant across all intervention conditions.

Finally, we interacted intervention condition (“Standard or Customized” *Yes = 1; No = 0*) with the personality dimensions across both time points to predict students’ belonging at the end of their first year. All other model specifications (including the use of student- and school-level covariates) are similar to the primary analyses. We find that moderations by intervention condition are nonsignificant in regard to all of the personality dimensions. These exploratory analyses were conducted in Stata/SE Version 15.1 [10].

A few notable results emerge (Table S6). First we find that across both time points the positive association between agreeableness and belonging is maintained. However the associations between (a) extraversion, (b) neuroticism, (c) conscientiousness, (d) openness and T_2_ belonging are less consistent and less precise.

### **Table S6: Moderations by intervention condition.**

|  | **T1 Personality Predicting T2 Belonging** | **T2 Personality Predicting T2 Belonging** |
| --- | --- | --- |
| **Extraversion** | .064  (.034) | .131***  (.024) |
| **Neuroticism** | -.055  (.036) | -.077**  (.026) |
| **Agreeableness** | .114**  (.044) | .184***  (.030) |
| **Conscientiousness** | .047  (.044) | .042  (.031) |
| **Openness** | -.026  (.037) | .010  (.027) |
| **Standard or Customized Condition** | -.535  (.367) | -.166  (.253) |
| **Extraversion x Standard or Customized Condition** | .064  (.042) | .052  (.029) |
| **Neuroticism x Standard or Customized Condition** | -.011  (.044) | -.005  (.031) |
| **Agreeableness x Standard or Customized Condition** | .058  (.054) | -.030  (.036) |
| **Conscientiousness x Standard or Customized Condition** | -.006  (.055) | .012  (.037) |
| **Openness x Standard or Customized Condition** | .051  (.046) | .020  (.032) |
| ***N* Observations** | 2,137 | 4,262 |

Standard errors in parentheses. The reference category is White, continuing-generation women in the control condition. To economize on space, we do not report the dummy variables for each school. Each column reports the results “Large Schools” coefficient from separate, covariate-adjusted, regressions where “Standard or Customized Condition” indicator is interacted with each of the personality dimensions to predicting T2 Belonging.

*p < .05. **p < .01. p*** < .001.

# **Robustness analyses using restricted inclusion criteria**

As noted in the main text, our primary analyses included all students who completed the personality scale at least once and completed the T_2_ belonging scale. There were a number of students who only completed the personality scale only once. This could be for one of three reasons. First, it could be that the personality questions were not included in the T_2_ survey at their school. (The decision of which questions to include where was made jointly by the original research team and the partner schools.) Second, it could be that although the personality questions were included in the T_2_ survey at the student’s school, the student was not included in the sample invited to participate in the survey. Third, it could be that although the personality questions were included in the T_2_ survey at the student’s school and the student was in the invited sample, the student chose not to answer the personality questions at either T_1_ or T_2_.

As a robustness check, we present the main manuscript analyses here with only those students who otherwise meet the inclusion criteria and completed the personality scales at both times (*N =* 1,646). For Research Question 1, the more restricted sample comprises 77% of the participants that were included in the manuscript, while for Research Question 2, the more restricted sample comprises only 39% of the participants who were included in the manuscript.

## **T_1_ personality and T_2_ belonging in restricted sample**

In our preferred model which controlled for school and personal characteristics, extraversion and agreeableness at the beginning of students’ first year were associated with their belonging at the end of their first year (Table S7). Specifically, T_1_ extraversion and T_1_ agreeableness were each positively associated with T_2_ belonging; the higher a student’s extraversion or agreeableness during the summer before college, the higher their sense of belonging at the end of their first year of college. This model explained 10.2% of the variance in T_2_ belonging (R^2^= .102, *F*(25, 1620) = 8.436, *p* < .001). In addition, students who identified racial-ethnically as Black, Asian, or “Other” reported lower T_2_ belonging compared to White students.

### **Table S7: T_1_ personality predicting T_2_ belonging in restricted sample.**

|  | **Personality-only** | | **Preferred** | | **Covariate-only** | |
| --- | --- | --- | --- | --- | --- | --- |
|  | ***B***  ***(SE)*** | ***95% CI*** | ***B***  ***(SE)*** | ***95% CI*** | ***B***  ***(SE)*** | ***95% CI*** |
| **Extraversion** | .138***  (.024) | [.092, .185] | .124***  (.023) | [.078, .170] | - | - |
| **Neuroticism** | -.026  (.024) | [-.073, .022] | -.047  (.025) | [-.096, .003] | - | - |
| **Agreeableness** | .120***  (.030) | [.061, .178] | .149***  (.029) | [.091, .207] | - | - |
| **Conscientiousness** | .089**  (.030) | [.030, .148] | .038  (.030) | [-.022, .097] | - | - |
| **Openness** | -.002  (.025) | [-.052, .047] | .017  (.025) | [-.033, .066] | - | - |
| **Black** | - | - | -.623***  (.087) | [-.794, -.452] | -.555***  (.088) | [-.728, -.382] |
| **Asian** | - | - | -.291**  (.090) | [-.468, -.113] | -.337***  (.092) | [-.517, -.156] |
| **Hispanic** | - | - | -.186  (.101) | [-.383, .011] | -.153  (.103) | [-.354, .049] |
| **Native** | - | - | -.435  (.279) | [-.982, .112] | -.414  (.286) | [-.975, .148] |
| **Multiracial** | - | - | -1.025  (.924) | [-2.837, .786] | -.688  (.945) | [-2.541, 1.165] |
| **Other Race** | - | - | -.304**  (.097) | [-.495, -.113] | -.313**  (.100) | [-.508, -.117] |
| **Unspecified Race** | - | - | .118  (.652) | [-1.162, 1.397] | -.041  (.669) | [-1.353, 1.272] |
| **Man** | - | - | -.008  (.054) | [-.114, .098] | -.008  (.053) | [-.112, .097] |
| **Other Gender** | - | - | -.182  (.189) | [-.552, .189] | -.308  (.192) | [-.685, .069] |
| **First-Generation Student** | - | - | -.025  (.055) | [-.133, .083] | -.031  (.056) | [-.141, .079] |
| **Unknown Generation Status** | - | - | -.171  (.270) | [-.700, .358] | -.228  (.276) | [-.769, .313] |
| **Adjusted R²** | 0.051 | | 0.102 | | 0.053 | |
| ***N* Observations** | 1,646 | | 1,646 | | 1,646 | |

The reference category is White, continuing-generation women in the control condition. To economize on space, we do not report the dummy variables for each school and condition (available from authors upon request).

*p < .05. **p < .01. ***p < .001.

Our preferred model that included the covariates in addition to personality had greater predictive validity than the covariate-only model (*F*(5, 1625) = 18.594, *p* < .001). Including personality in the model explained an additional 4.9% of the variance in students’ T_2_ belonging over and above the covariates.

## **T_2_ personality and T_2_ belonging in restricted sample**

In our preferred model which controlled for school and personal characteristics, extraversion, agreeableness, and neuroticism at the end of students’ first year were associated with their belonging at the end of their first year (Table S8). Specifically, T_2_ extraversion and T_2_ agreeableness (*b* = .091, *se* = .028, *t* = 3.276, *p* = .001) were each positively associated with T_2_ belonging; the higher a student’s extraversion or agreeableness at the end of their first year of college, the higher their sense of belonging at that same time. In contrast, T_2_ neuroticism was negatively associated with T_2_ belonging; the lower a student’s neuroticism at the end of their first year of college, the higher their sense of belonging at that same time. This model explained 15.2% of the variance in T_2_ belonging (R^2^= .152, *F*(25, 1620) = 12.75, *p* < .001). In addition, students who identified racial-ethnically as Black, Asian, or “Other” reported lower T_2_ belonging compared to White students.

### **Table S8: T_2_ personality predicting T_2_ belonging in restricted sample.**

|  | **Personality-only** | | **Preferred** | | **Covariate-only** | |
| --- | --- | --- | --- | --- | --- | --- |
|  | ***B***  ***(SE)*** | ***95% CI*** | ***B***  ***(SE)*** | ***95% CI*** | ***B***  ***(SE)*** | ***95% CI*** |
| **Extraversion** | .200***  (.023) | [.155, .245] | .197***  (.023) | [.153, .242] | - | - |
| **Neuroticism** | -.065**  (.023) | [-.111, -.019] | -.074**  (.024) | [-.121, -.026] | - | - |
| **Agreeableness** | .152***  (.026) | [.100, .204] | .156***  (.026) | [.105, .207] | - | - |
| **Conscientiousness** | .091**  (.028) | [.036, .145] | .051  (.028) | [-.004, .106] | - | - |
| **Openness** | .009  (.025) | [-.039, .057] | .012  (.025) | [-.036, .061] | - | - |
| **Black** | - | - | -.600***  (.085) | [-.766, -.434] | -.555***  (.088) | [-.728, -.382] |
| **Asian** | - | - | -.297***  (.088) | [-.469, -.124] | -.337***  (.092) | [-.517, -.156] |
| **Hispanic** | - | - | -.151  (.098) | [-.342, .041] | -.153  (.103) | [-.354, .049] |
| **Native** | - | - | -.307  (.271) | [-.839, .226] | -.414  (.286) | [-.975, .148] |
| **Multiracial** | - | - | -1.134  (.898) | [-2.896, .627] | -.688  (.945) | [-2.541, 1.165] |
| **Other Race** | - | - | -.338***  (.094) | [-.523, -.152] | -.313**  (.100) | [-.508, -.117] |
| **Unspecified Race** | - | - | .464  (.635) | [-.781, 1.709] | -.041  (.669) | [-1,353, 1.272] |
| **Man** | - | - | .006  (.053) | [-.098, .110] | -.008  (.053) | [-.112, .097] |
| **Other Gender** | - | - | -.034  (.183) | [-.393, .326] | -.308  (.192) | [-.685, .069] |
| **First-Generation Student** | - | - | -.047  (.053) | [-.152, .058] | -.031  (.056) | [-.141, .079] |
| **Unknown Generation Status** | - | - | -.181  (.261) | [-.694, .331] | -.228  (.276) | [-.769, .313] |
| **Adjusted R²** | 0.106 | | 0.152 | | 0.053 | |
| ***N* Observations** | 1,646 | | 1,646 | | 1,646 | |

The reference category is White, continuing-generation women in the control condition. To economize on space, we do not report the dummy variables for each school and condition (available from authors upon request).

*p < .05. **p < .01. ***p < .001.

Our preferred model that included the covariates in addition to personality had greater predictive validity than the covariate-only model (*F*(5, 1625) = 38.768, *p* < .001). Including personality in the model explained an additional 9.8% of the variance in students’ T_2_ belonging over and above the covariates.

## **Restricted sample compared to manuscript sample**

These supplemental results analyzing only those participants who answered all of the questions relevant to our study show similar results to those reported in our manuscript.

### **Similarities**

In the analyses regarding the association between T_1_ personality and T_2_ belonging, we find that both T_1_ extraversion and T_1_ agreeableness are positively associated with T_2_ belonging in both the manuscript sample and the restricted sample. Additionally, in both samples, we find that the assocation between T_1_ conscientiousness and T_1_ openness with T_2_ belonging is nonsignificant.

In the analyses focused on the association between T_2_ personality and T_2_ belonging, we find that T_2_ extraversion and T_2_ agreeableness are both positively associated with T_2_ belonging, T_2_ neuroticism is negatively associated with T_2_ belonging, and the relationship between T_2_ openness and T_2_ belonging is nonsignificant in both the manuscript sample and the restricted sample.

### **Differences**

Only one difference appeared between the T_1_ personality and T_2_ belonging analyses reported in the main manuscript and those reported here. In the manuscript, we find a statistically significant negative association between T_1_ neuroticism and T_2_ belonging (*b* = -.060, *se* = .022, *t* = -2.722, *p* = .007), while in our supplemental results reported here, we find the relationship between T_1_ neuroticism and T_2_ belonging to be nonsignificant at the conventional significance levels (*b* = -.047, *se* = .025, *t* = -1.849, *p* = .065).

The only difference between the T_2_ personality and T_2_ belonging analyses reported in the main manuscript and those reported here concerns T_2_ conscientiousness. In the manuscript, we find a statistically significant positive association between T_2_ conscientiousness and T_2_ belonging (*b* = .048, *se* = .018, *t* = 2.655, *p* = .008). Alternatively, in our supplemental results reported here, we find a marginal effect such that T_2_ conscientiousness is positively associated with T_2_ belonging (*b* = .051, *se* = .028, *t* = 1.834, *p* = .067).

These lower significance values reported in the supplement may be due to slightly lower sample size and resulting statistical power. However, the magnitude of the coefficients and the patterns between the baseline and covariate-adjusted models are qualitatively similar when compared to main manuscript results overall.

# **References**

1. Gonzalez R, Griffin D. The correlational analysis of dyad-level data in the distinguishable case. Personal Relationships. 1999;6(4):449–69.
2. R Core Team. R: A language and environment for statistical computing [Internet]. Vienna, Austria; 2021. Available from: [https://www.R-project.org/](https://www.r-project.org/)
3. Stieger M, Flückiger C, Rüegger D, Kowatsch T, Roberts BW, Allemand M. Changing personality traits with the help of a digital personality change intervention. Proc Natl Acad Sci USA. 2021 Feb 23;118(8):e2017548118.
4. Roberts BW, Luo J, Briley DA, Chow PI, Su R, Hill PL. A systematic review of personality trait change through intervention. Psychological Bulletin. 2017 Feb;143(2):117–41.
5. Allemand M, Flückiger C. Personality change through digital-coaching interventions. Curr Dir Psychol Sci. 2022 Feb 1;31(1):41–8.
6. Walton GM, Murphy MC, Logel C, Yeager DS, Goyer JP, Brady ST, et al. Where and with whom does a brief social-belonging intervention promote progress in college? Science. 2023 May 5;380(6644):499–505.
7. Brady ST, Muragishi GA, Getu R. The unsettled questions of belonging uncertainty. [Manuscript submitted for publication]. Department of Psychology, Wake Forest University.
8. Gopalan M, Linden-Carmichael A, Lanza S. College students’ sense of belonging and mental dealth amidst the COVID-19 pandemic. Journal of Adolescent Health. 2022 Feb 1;70(2):228–33.
9. Blodorn A, Brady ST, the College Transition Collaborative (CTC). Social-belonging multi-site randomized control trial: Technical summary [Internet]. 2018 Mar 28 [cited 2023 Jun 26]. Available from:<https://docs.google.com/document/d/e/2PACX-1vQGerROOs_qSOr_uugGeR9Pm5xLidVy-zyTY_BfIw-mno5Nv2aDs9tJcdH05i1_wP6YvKz5b2Ezb_WO/pub>
10. StataCorp. Stata statistical software: Release 15. College Station, TX: StataCorp LLC; 2017.
