# Supplementary material for: Who feels like they belong? Personality and belonging in college
Source: PLoS One. 2024 Jan 17;19(1):e0295436. doi: 10.1371/journal.pone.0295436 (PMC10793887; doi:10.1371/journal.pone.0295436)
Supplement: Attachment — Submitted filename: Response to Reviewers.docx [file pone.0295436.s002.docx]

November 18, 2023

Dear Dr. Mustafa:

Thank you for your invitation to resubmit our manuscript “Who feels like they belong? Personality and belonging in college” [PONE-D-23-21304], which examines the relationship between personality and belonging during the transition to college, a critical time in students’ academic trajectories. We are grateful to you and the reviewers for your thoughtful consideration of our work and your helpful suggestions.

In the sections that follow, we outline the suggestions made in this round of review and our responses to them. In our responses, we indicate the specific changes we made and, as appropriate, point to relevant pages and lines in the updated manuscript. Furthermore, we highlight all additions and use track changes to document any deletions in a marked-up copy of our manuscript. It is worth noting the largest change made to our original version: removing our analyses concerned with the relationship between personality change and belonging, as recommended by Reviewer 1.

Again, we are grateful for the opportunity to resubmit a revised version of this manuscript to PLOS ONE. We hope that you will now find it acceptable for publication.

Sincerely,

Alex Stubblebine

Maithreyi Gopalan

Shannon Brady

***Responses to Editor’s Comments***

**When submitting your revision, we need you to address these additional requirements.**

1. ***Please ensure that your manuscript meets PLOS ONE’s style requirements, including those for file naming.***

We appreciate the Editor calling our attention to this. We have gone through our manuscript and have ensured that it meets PLOS ONE’s style requirements.

2. ***Please include your tables as part of your main manuscript and remove the individual files. Please note that supplementary tables (should remain/ be uploaded) as separate “supporting information” files.***

The appropriate tables have been incorporated as part of our main manuscript. The supplementary tables have been embedded into our Supplemental Material and uploaded to PLOS ONE as “supporting information” files.

***Responses to Comments by Reviewer 1***

1. **Concern regarding the examination of personality change.**
   *“Although this was a well-written manuscript, my main concern was the examination of the so-called "personality change” (p. 10). Because of the different test-taking contexts, I would argue that the responses to the personality scale cannot be compared between the two time periods. Pages 11-12 explain that students first completed the personality scale before attending university/college. The second session was, “At the end of their first year” (p. 12). The students will be in completely different mind sets for each test-taking time. Time 1 would most likely reflect personality plus eustress or excitement/concern about starting school. Time 2 is probably reflecting personality plus distress because the end of term is either during or possibly at the start of final exams. These differences in context will clearly influence how individuals will respond to the personality items. For example, if anxious about exams, individuals may endorse more neuroticism items and fewer agreeableness items. I would therefore suggest that all mention of “personality change” be removed from the manuscript, especially the analyses and text starting at the bottom of page 16 and Table 5.”*

Thank you for highlighting this concern about the personality change measure. We acknowledge that personality measures could be responsive to contextual differences such as state anxiety induced by exam season [1]. It may be true that the stress of starting school and distress of exams would impact how individuals respond to personality items. However, given that there are no other analyses in the current literature which examine the relationship between personality change and belonging, we wanted to make these results available to others. Therefore, we have decided to address this comment by removing all mention of “personality change” from the manuscript and including the relevant analyses in the Supplemental Material instead.

2. **Clarification on the contents of Table 6.**
   *“The text on page 18 states that Table 6 is the mean differences but Table 6 lists regression weights (not the right values).”*

Thank you for catching this error. We have edited the text to accurately describe the table’s contents (page 23, line 391).

3. **Minor typos.**

*“Page 9, line 181, I believe it should be “with general” and not “to”.”*

We have made the suggested edit on page 9, line 184.

*“Page 13, line 276, the alpha symbol is missing (might be easier to simply use the word, “alpha”).”*

We have made sure to include the alpha symbol in our resubmission on page 15, line 286.

4. **Refer to tables differently.**
   *“Results section – it is a bit awkward to simply have, for example, “See Table 3.” in the text. Simply put into brackets and connect to the preceding sentence.”*

Thank you for this efficient suggestion. We have implemented it.

***Responses to Comments by Reviewer 2***

5. **Comment on strengths and limitations of paper.**
   *“The differential impact of students' personalities on their sense of belonging is a relevant extension of existing research on students' sense of belonging. The authors had access to a really large number of students to answer their research questions. A closer look at the manuscript revealed that the paper has several strengths, but also some issues that the authors should address and discuss prior to publication.”*

Thank you for recognizing the contributions and strengths of our manuscript. We hope that our edits and responses sufficiently address your comments and concerns.

6. **Upload scripts as scripts.**

*“I appreciate the authors' efforts to make their data and scripts openly available. However, I wondered why they were copied into a Word document and not uploaded as a script.”*

Thank you for your recognition of our openly available data and scripts. We have addressed this comment by uploading the relevant files as scripts rather than Word documents. These files can be found on OSF (<https://osf.io/mv6ek/>).

7. **Edit or drop last sentence of the abstract.**
   *“The last sentence of the summary is not very informative - either provide a specific impact or drop the sentence.”*

Thank you for pointing out how we could strengthen our abstract. We believe that our discussion of the implications for future research and practice is an important component of our paper. Therefore, we have decided to edit the sentence to include the specific impacts on page 2, lines 38-40.

8. **Add references.**

*“P3: ‘Students’ feelings of belonging may be especially important during academic transitions, such as the first year of college, when students are navigating new challenges for the first time.’ I think this statement needs a reference.”*

*“P3, l. 54: Also, extraverts, seem to suffer more when they cannot pursue their inherent urge for social contact, see e.g., Weiß, M., Rodrigues, J., & Hewig, J. (2022). Big five personality factors in relation to coping with contact restrictions during the COVID-19 pandemic: A small sample study. Social Sciences, 11(10), 466.”*

*“P8, l. 175: Also, in terms of social media use, which is a way for most students to "belong.", see e.g., Weiß, M., Baumeister, H., Cohrdes, C., Deckert, J., Gründahl, M., Pryss, R., & Hein, G. (2022). Extraversion moderates the relationship between social media use and depression. Journal of Affective Disorders Reports, 8, 100343.”*

Thank you for making these suggestions and in particular for pointing us toward specific papers. We added a reference [2] to support the statement about transitions on page 3, line 50. With regard to the ***Weiß*** citations, we have edited page 3, line 55 and page 8, lines 177-178. to effectively make use of this citation to support our introduction.

9. **Streamline final paragraphs of the introduction.**
   *“Overall, I like the introduction very much, but it could be streamlined to some extent. In the last paragraphs (p. 10), I think the authors mix research questions and hypotheses a bit. A clearer division (especially regarding the hypotheses) would be helpful.”*

Thank you for calling our attention to this. We carefully read the manuscript with an eye for streamlining and made several edits. We have rearranged the contents of mentioned paragraphs in an effort to improve clarity. Specifically, we have moved the paragraph regarding our exploratory analyses to immediately follow the paragraph describing our primary analyses and precede the paragraph introducing our hypotheses. Lastly, we have added subheadings titled “Research questions” (page 9, line 203), “Hypotheses” (page 10, line 212), and “Contributions” (page 10, line 216) to the relevant paragraphs to distinguish and make the divisions between research questions and hypotheses clearer.

10. **Request to include the results including only participants who answered all of the questions of our study.**

*“P12 (participants): Wouldn't it be preferable for the interpretation of the results to include only those who answered all the questions? I understand that the numbers deviate heavily. Could the authors at least test whether the results would differ if only the students who answered all questions were analyzed?”*

Thank you for this suggestion. In the manuscript, we chose to interpret the results of those who (a) were a first-year (non-transfer) student, (b) completed the personality scale at least once, and (c) completed the belonging scale in the survey at the end of their first year in order to increase the statistical power of our study. Additionally, by including the aforementioned participants in each analysis, we were able to more accurately represent the student population that would benefit from our study.

It was not obvious to us why it would have been preferable to restrict the sample to only those who have data for all of the variables under investigation. Certainly, doing so keeps participants consistent across analyses, but also substantially reduces sample size. That said, we do think this is a worthwhile robustness check, so we have conducted these analyses testing whether the results would differ if only the students who answered the personality scale at both Time 1 (T_1_) and Time 2 (T_2_) and the belonging scale at T_2_ were included. These analyses are reported in the Supplemental Material.

The selective sample that was used in the supplement consisted of 1,646 participants. For Research Question 1, the more restricted sample comprises 77% of the participants that were included in the manuscript, while for Research Question 2, the more restricted sample comprises 39% of the participants who were included in the manuscript.

The results for the restricted sample exhibit similar relationships to those reported in the manuscript regarding every personality trait at both time points except for T_1_ neuroticism and T_2_ conscientiousness. The results for T_1_ neuroticism and T_2_ conscientiousness decrease in significance, however the magnitude and direction of their association with T_2_ belonging are qualitatively similar to the results reported in the main manuscript.

11. **Address low reliabilities as a limitation.**
    *“P13: There are some very low reliabilities in these questionnaires. The authors should address this as a limitation.”*

In our original manuscript, we mentioned in the Methods that shorter measures typically have lower reliability [4]. We also provided references showing that our reported reliability is in line with previous research using the Big Five Inventory-10 [BFI-10; 3, 5-7]. However, we did not specifically address the low reliabilities as a limitation in our Discussion. We now elaborate on this in our “Strength, Limitations, and Future Directions” section on page 27, lines 479-495. In particular, we explicitly state, “A second consequence of using such a brief measure of personality is that the reliabilities for the dimensions were not high.”

12. **Provide transparency regarding exploratory analyses.**

*“P14: I think it's fine not to include the pre-registered exploratory analyses in the main paper. But why not in a supplement? Or are the results so interesting that the authors are planning a separate paper? I would like to see more transparency here.”*

Truthfully, we had not ever run the analyses. We began with the analyses that most interested us and, as we progressed, decided that the preregistered exploratory analyses were beyond the scope of what we thought we could effectively present in one manuscript. However, we could see how the inclusion of these results would allow us to be more transparent and informative. Therefore, we have run the analysis examining whether intervention condition moderates effects and the regression analyses, similar to the primary analyses, with belonging uncertainty as the outcome. The results from both of these analyses can be found in our Supplemental Material.

13. **Edit the subheadings in the Results to be more direct.**
    *“The subheadings (e.g., Is Students’ Personality at the Beginning of College Predictive of Their Belonging at the End of Their First Year?) read like undirected hypotheses, which is confusing (at least to me) – I would prefer statements. Also, I personally find the "yes" after each of these questions odd. You want to confirm or reject hypotheses, not have a dialogue with your subheadings.”*

As suggested, we have rewritten the subheadings in this section of our manuscript to be more direct and have removed the dialogue from the text. For example, the subheading that previous read “Is Students’ Personality at the Beginning of College Predictive of Their Belonging at the End of Their First Year?” currently reads as “Associations between students’ personality at the beginning of college and their belonging at the end of first year”.

14. **Add figures to depict most important results.**

*“Why did the authors not present any figures at all? I think some visualizations for the most important results would help the reader.”*

We appreciate this question. We, too, often prefer visualizations in manuscripts. However, given that this manuscript focuses on the associations between variables (rather than, for example, condition differences) we believe that tables are the most effective means of communicating our findings. Our use of tables is consistent with other studies in the literature examining the relationships between perceived and actual personality change and between personality and academic achievement [8, 9].

15. **Include test-retest reliability.**
    *Could the authors provide test-retest reliability for the Big Five traits? With the given sample size this would be a nice additional information for interested readers.”*

We are happy to provide test-retest reliability for the Big Five traits in the manuscript. It is now included in our section titled “Personality.” on page 15, lines 280-282.

**References**

1. Reich J, Noyes R, Coryell W, O’Gorman T. The effect of state aniety on personality measurement. The American journal of psychiatry. 1986 Jul 1;143:760–3.
2. Meehan C, Howells K. ‘What really matters to freshers?’: evaluation of first year student experience of transition into university. Journal of Further and Higher Education. 2018 Oct 3;42(7):893–907.
3. Thalmayer AG, Saucier G, Eigenhuis A. Comparative validity of Brief to Medium-Length Big Five and Big Six Personality Questionnaires. Psychological Assessment. 2011;23(4):995–1009.
4. Duckworth AL, Yeager DS. Measurement Matters: Assessing Personal Qualities Other Than Cognitive Ability for Educational Purposes. Educational Researcher. 2015 May 1;44(4):237–51.
5. Balgiu BA. The psychometric properties of the Big Five inventory-10 (BFI-10) including correlations with subjective and psychological well-being. Global Journal of Psychology Research: New Trends and Issues. 2018 Jun 18;8(2):61–9.
6. Credé M, Harms P, Niehorster S, Gaye-Valentine A. An evaluation of the consequences of using short measures of the Big Five personality traits. Journal of Personality and Social Psychology. 2012;102(4):874–88.
7. Carciofo R, Yang J, Song N, Du F, Zhang K. Psychometric Evaluation of Chinese-Language 44-Item and 10-Item Big Five Personality Inventories, Including Correlations with Chronotype, Mindfulness and Mind Wandering. PLOS ONE. 2016 Feb 26;11(2):e0149963.
8. Robins RW, Noftle EE, Trzesniewski KH, Roberts BW. Do People Know How Their Personality Has Changed? Correlates of Perceived and Actual Personality Change in Young Adulthood. J Personality. 2005 Apr;73(2):489–522.
9. Steinmayr R, Weidinger AF, Wigfield A. Does students’ grit predict their school achievement above and beyond their personality, motivation, and engagement? Contemporary Educational Psychology. 2018 Apr 1;53:106–22.
